# Reversible rearrangement of magnetic nanoparticles in solution studied using time-resolved SAXS method

**DOI:** 10.1107/S1600577519004909

**Published:** 2019-06-11

**Authors:** Lanqing Huang, Jingeng Mai, Qihui Zhu, Zhen Guo, Siying Qin, Peilin Yang, Xuanxuan Li, Yingchen Shi, Xiaotian Wang, Qining Wang, Na Li, Can Xie, Haiguang Liu

**Affiliations:** a Complex Systems Division, Beijing Computational Science Research Center, 8 E Xibeiwang Rd, Haidian, Beijing 100193, People’s Republic of China; b The Robotics Research Group, College of Engineering, Peking University, 5 Yiheyuan Rd, Haidian, Beijing 100871, People’s Republic of China; c State Key Laboratory of Membrane Biology, Laboratory of Molecular Biophysics, School of Life Sciences, Peking University, Beijing 100871, People’s Republic of China; d Department of Engineering Physics, Tsinghua University, Beijing 100086, People’s Republic of China; e National Facility for Protein Science in Shanghai, Zhangjiang Lab, 333 Haike Road, Shanghai 201204, People’s Republic of China; f Interdisciplinary Science Division, Beijing Computational Science Research Center, 8 E Xibeiwang Rd, Haidian, Beijing 100193, People’s Republic of China

**Keywords:** superparamagnetic nanoparticles, small-angle X-ray scattering, magnetic fields, dynamic assembly, monodispersed states, time-resolved dynamics

## Abstract

Superparamagnetic nanoparticles have broad applications in biology and medicines. Quantitative measurements of magnetic beads in solution are essential in gaining comprehensive understanding of their dynamics and developing applications. Here, using synchrotron X-ray sources combined with well controlled magnetic fields, the results from small-angle X-ray scattering (SAXS) experiments on superparamagnetic particles in solution under the influence of external magnetic fields are reported. The particles mostly remain in monodispersed states and the linear aggregates tend to be aligned with the external magnetic field. After removing the magnetic fields, the superparamagnetic nanoparticles quickly recover to their original states indicating high reversibility of the rearrangement under the control of a magnetic field. The external magnetic field instrument composed of paired permanent magnets is integrated into the SAXS beamline at the Shanghai Synchrotron Radiation Facility providing a platform for studying time-resolved dynamics induced by magnetic fields.

## Abstract 2

Magnetic field induced rearrangement of magnetic nanoparticles was observed using synchrotron small-angle X-ray scattering methods.

## 1. Introduction   

Superparamagnetic nanoparticles (SMNPs) are magnetic particles that are sufficiently small to be considered as composed of single magnetic domains, such that an external magnetic field (MF) can magnetize the particles with high magnetic susceptibility (Akbarzadeh *et al.*, 2012 ▸). In the absence of an external MF, the magnetization of such nanoparticles can randomly flip directions because of thermal fluctuations at finite temperatures. These nanoparticles have important applications in biological and biomedical sciences, such as controlled drug delivery (Gupta & Wells, 2004 ▸; Neuberger *et al.*, 2005 ▸; Xu & Sun, 2013 ▸), cell/tissue labelling or imaging contrast enhancer (Chouly *et al.*, 1996 ▸; Thorek *et al.*, 2006 ▸), and reaction catalysis (Fan & Gao, 2006 ▸; Akbarzadeh *et al.*, 2012 ▸; Dalpozzo, 2015 ▸). On the other hand, nanotoxicity is also under active study to fully understand the health issues raised by nanoparticles (Elsaesser & Howard, 2012 ▸; Bahadar *et al.*, 2016 ▸). This research converges to the quantitative studies of SMNP arrangements and dynamics in solution, especially under the influence of an external MF.

**Figure 1:**
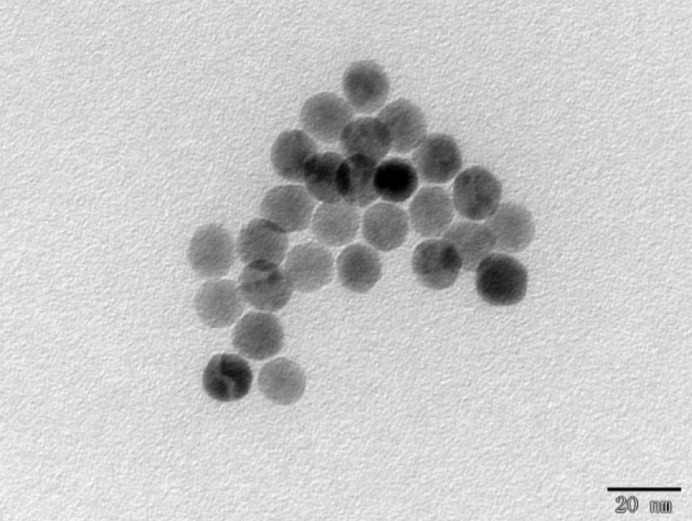
The synthetic iron oxide nanoparticles. The particles are nearly spherical with a mean radius of about 6.5 nm under electron microscope.

**Figure 2:**
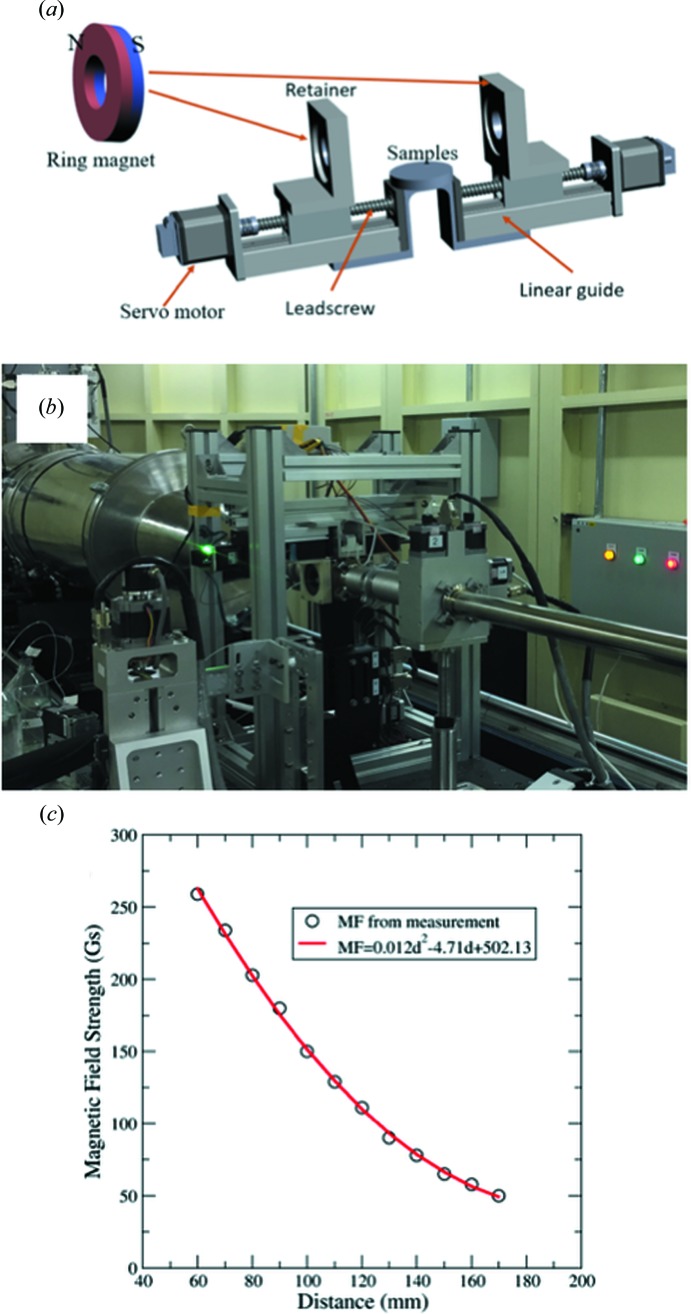
Experimental setup. (*a*) Schematic drawing of the MF instrument, where the MF is provided by a pair of permanent magnets mounted on a supporting rod. (*b*) The instrument is installed at the SSRF 19U2 beamline. The MF at the sample position is horizontal and perpendicular to the incidence X-ray beam. (*c*) The MF strength is controlled by adjusting the distance between the ring magnets. The analytical formula [MF = (0.012*d* ^2^ − 4.71*d*) + 502.13] is obtained by non-linear fitting (*R* ^2^ = 0.9990).

The physical properties and morphology characteristics of SMNPs can be obtained using several experimental methods. Scanning/transmission electron microscopy (STEM) can be used to record high-resolution images of SMNPs, to reveal the shape and size information (Jadzinsky *et al.*, 2007 ▸; Buhr *et al.*, 2009 ▸; Reddy *et al.*, 2012 ▸) (see Fig. 1 ▸). Although it is possible to probe the the state of SMNPs in solution using STEM, the experiments are very challenging (Lee *et al.*, 2017 ▸). Furthermore, probing the dynamics of SMNPs under the influence of an external MF is often limited by instruments. The treatment of SMNP samples for STEM measurements may result in changes in SMNP states when compared with that in bulk solvent. For example, the SMNPs could become more concentrated and form clusters on the sample support if solvent is reduced, even in the case where sample solution is sealed within thin protection layers. On the contrary, dynamic light scattering or small-angle X-ray scattering (SAXS) can be applied to probe the properties of SMNPs in solution (Jans *et al.*, 2009 ▸; Nobbmann & Morfesis, 2009 ▸; Partyka-Jankowska *et al.*, 2014 ▸; Li *et al.*, 2016 ▸; Szczerba *et al.*, 2017 ▸). Although there have been extensive applications of synchrotron radiation in the study of magnetism (Dürr *et al.*, 2009 ▸), time-resolved experiments were not reported, to the best of our knowledge. In this study, SAXS experiments were carried out to study the dynamics of SMNPs in a time-resolved approach, in combination with a controlled external MF that is provided using a pair of permanent magnets. The sample was placed in the centre region in the space between the two magnets (Fig. 2 ▸). By varying the distance between these two magnets, the strength of the MF was controlled precisely. The MF strength can be further changed by using magnets with different remanence fields. The external MF strength dependence on the magnet distance was calibrated by a magnetometer [Fig. 2 ▸(*c*)].

In the time-resolved study, the SAXS profiles were measured once per second while varying the distance between the two magnets. The experimental data reveal that the SMNPs respond to the external MF rapidly on a time scale faster than that accessible in this measurement. Furthermore, the change induced by the MF is reversible, *i.e.* the SMNPs tend to recover their original states after removing the external MF. The integration of MF instruments to synchrotron SAXS beamlines allows direct measurement of the dynamics of SMNPs in solution at controlled temperatures, providing a platform for quantitative analysis of SMNP properties.

## 2. Methods and materials   

### 2.1. SMNPs   

The SMNPs used in this experiment are synthesized by BeaverBeads Inc., Suzhou, China. The nominal diameter of the iron oxide SMNP (γ-Fe_2_O_3_) was designed to be 13.0 nm, with a coating layer of glycan molecules. The synthesized SMNPs were examined and characterized using STEM, as shown in Fig. 1 ▸. In solution, the SMNPs remain in a monodispersed state at a concentration of 1 mg ml^−1^ (about 10^14^ particles ml^−1^).

### 2.2. MF instrument and integration into SAXS beamlines   

A MF instrument was customized with a distance-regulation platform and a pair of permanent magnets so that the instrument was fully compatible with the setups at beamline 19U2 at the Shanghai Synchrotron Radiation Facility (SSRF). The permanent magnets used in this study were grade N52 Nd–Fe–B magnets with ring shapes, each with an inner diameter of 50 mm, an outer diameter of 70 mm and a thickness of 6 mm. The residual induction of one magnet is 2000 G. The distance-regulation platform consisted of magnet re­tainers, servo motors, leadscrews and linear guides [see Fig. 2 ▸(*a*)]. Driven by servo motors, the retainers can move to desired positions along the guide rail at controlled speeds in both directions. In order to produce stable magnet fields, two annular permanent magnets were installed to retainers symmetrically along a common axis and experiment samples were placed in the central region of a nearly uniform MF. The two facing poles are opposite so that the MF is pointing from the N-pole of one magnet to the S-pole of the other magnet. Note that the diameter of the capillary (1.5 mm) is much smaller than the diameter of the magnets (outer diameter = 70 mm). The sample being measured was placed in the uniform MF that is perpendicular to the X-ray incidence direction (see Fig. 2 ▸). When the distance between the two magnets was adjustable in a range from 50 mm to 170 mm, correspondingly, the MF strength of the sample region could be changed from 250 G to 50 G. The annular permanent magnets can be installed or removed from the magnet re­tainers quickly if needed.

The MF strength was calibrated using a magnetometer sensor (BST-201 Tesla Meter from Beiyi) to a precision of 1.0 G. The MF strength dependence on distance between magnets can be approximately described with a quadratic function in this distance range [see Fig. 2 ▸(*c*)]. The MF instrument is designed to be flexible to accommodate different sample environments, such as vacuum box, temperature-control platform, stop-flow devices, *etc*.

### 2.3. SAXS experiment and data collection   

SAXS experiments were performed at beamline BL19U2 of the National Center for Protein Science Shanghai (NCPSS) at the SSRF. The wavelength, λ, of the X-ray radiation was set as 1.033 Å. Scattered X-ray intensities were collected using a Pilatus 1M detector (DECTRIS Ltd, Baden, Switzerland). The sample-to-detector distance was set such that the detecting range of momentum transfer [*q* = 4πsin(θ)/λ, where 2θ is the scattering angle] of SAXS experiments was 0.008–0.4 Å^−1^. The SMNP sample was kept in pure water, the data collection was carried out at room temperature (∼25°C). The X-rays were focused to the detector plane with a focal size of 0.40 mm × 0.06 mm (H × V) at the sample position with a photon flux of 5 × 10^12^ photons s^−1^. A 1% attenuator was applied to reduce the radiation damage and to protect the detector. The SMNP sample was loaded onto the quartz capillary with a diameter of 1.5 mm and a wall thickness of 10 µm. Because the induced dynamics and rearrangement of SMNPs might be affected by the buffer flow, the flowing of the buffer was stopped after each sample loading. During the measurement for each sample, the SMNP buffer was kept still inside the capillary until the next sample loading. This provided a buffer environ­ment to allow the SMNP to respond to the external MF by removing the interference of other factors, such as turbulence or shearing force. The exposure time was set to be 1 s for each measurement.

To study the rearrangement dynamics of SMNPs in the varying MF, the data collection was accomplished in the automatic data-collection mode. The MF variation cycle starts with one configuration that corresponds to the weak MF (50 G), moving to the configuration that provides the strong MF (250 G), and back to the original configuration. The SAXS profile of buffer was measured and subtracted to obtain the sample SAXS profiles. The SAXS profiles for SMNP solution was also measured without applying an external MF (except the earth’s MF, which is ∼0.5 G at the experimental site).

### 2.4. Data analysis and modelling   

#### 2.4.1. Particle size distribution analysis   

By assuming the SMNP to be of a spherical shape, the size distribution is estimated by fitting experimental data with theoretical SAXS profiles of spheres with various radii. The theoretical SAXS profile for a sphere with radius *R* is given by the following equation,
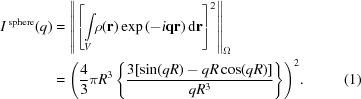
Here, ρ(**r**) is the electron density at position **r**, *q* = |**q**| is the modulus of momentum transfer **q**, and ∥…∥_Ω_ is the rotational averaging operation.

The size distribution function was obtained by fitting to the experimental data 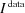 using a set of SAXS profiles (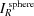) of different-sized spheres, *i.e.* optimizing the percentage [*w*(*R*)] of each component to obtain the minimum 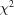,
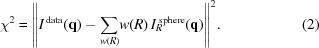
The weighting factors, *w*(*R*), were regularised by a Gaussian distribution to avoid overfitting. The optimization problem becomes finding the mean and σ of the Gaussian distribution.

#### 2.4.2. Modelling of SMNP assemblies   

Because the SMNPs interact with each other weakly, we assume that the percentage of assemblies is low and can be represented using a two-bead-chain (TBC) model composed of two SMNPs stuck together in a linear chain shape. Because the induced magnetization is aligned with the external MF, the 2D scattering patterns were simulated for the TBC model in the orientation where the major axis of the TBC model is perpendicular to the incidence X-ray direction. The experimental 2D scattering patterns (not 1D profiles) were fitted using simulated data from a single SMNP and the TBC model. The intensity values were calculated using the direct sum­mation of the scattered wavefunctions. Considering that some TBC particles may not be perfectly aligned with the MF, the scattering patterns from randomly oriented TBC particles were also simulated to generate an average pattern with angular isotropic intensities. A similar cost function as equation (2) was minimized to obtain the optimal parameters
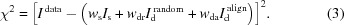
Again, 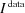 is the experimental 2D pattern for samples with multiple components, dominated by monodispersed spherical particles and their assemblies represented with the TBC model. The monodispersed SMNP has a scattering intensity pattern of *I*_s_, and the weighting factor is denoted as *w*_s_. The TBC contributions were from two parts, samples that are randomly oriented and those that are aligned with MF, whose corresponding scattering patterns are denoted as 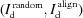 with associated weighting factors 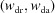, respectively. The simulation patterns were generated using a direct summation approach from dummy atoms filled in a sphere or in the TBC model. The average intensity of scattering patterns from 1000 randomly oriented TBC particles were used to represent the 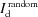. The intensity of three patterns were scaled such that the *I*(*q* = 0) has a ratio of 1:4:4, to match the desired electron numbers in each case.

## 3. Results   

### 3.1. SMNP size distributions without external MFs   

**Figure 3:**
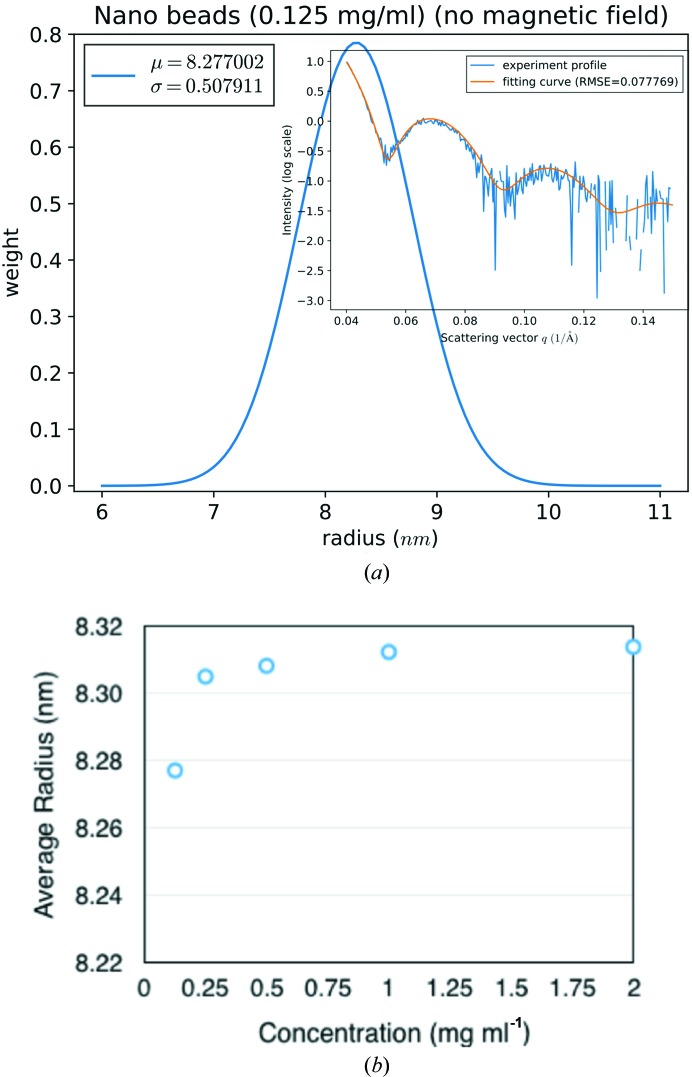
Nanoparticle size distributions. (*a*) The size distribution determined from SAXS profile for SMNPs at a concentration of 0.125 mg ml^−1^ without an external MF. The inset shows the fitting to experimental data. (*b*) The average size of SMNPs at five different concentrations (0.125 mg ml^−1^ to 2.0 mg ml^−1^). The width of the Gaussian distribution is approximately 0.5 nm for all five concentrations.

The synthesized SMNP was designed to have a radius of 6.5 nm, excluding the coating glycan molecules. Using the theoretical profiles of spherical objects [equation (1)], the optimal fitting was obtained with spheres with an average radius of ∼8.30 nm (Fig. 3 ▸). The estimated average sizes exhibited a slight dependency on the concentration. The variations are well within one standard deviation of the assumed Gaussian distributions. The increase from 8.27 nm at 0.125 mg ml^−1^ to 8.31 nm at 2.0 mg ml^−1^ might be because of the increased aggregation/assembly at higher concentrations. The size is consistently larger than the results from electron-microscopy imaging. The difference could be attributed to two sources: (1) the coating glycan molecules were not well observed in electron microscopes because of the low contrast and (2) the existence of SMNP aggregates or assembly in solution can result in larger apparent sizes.

### 3.2. Reversible rearrangement of SMNPs   

**Figure 4:**
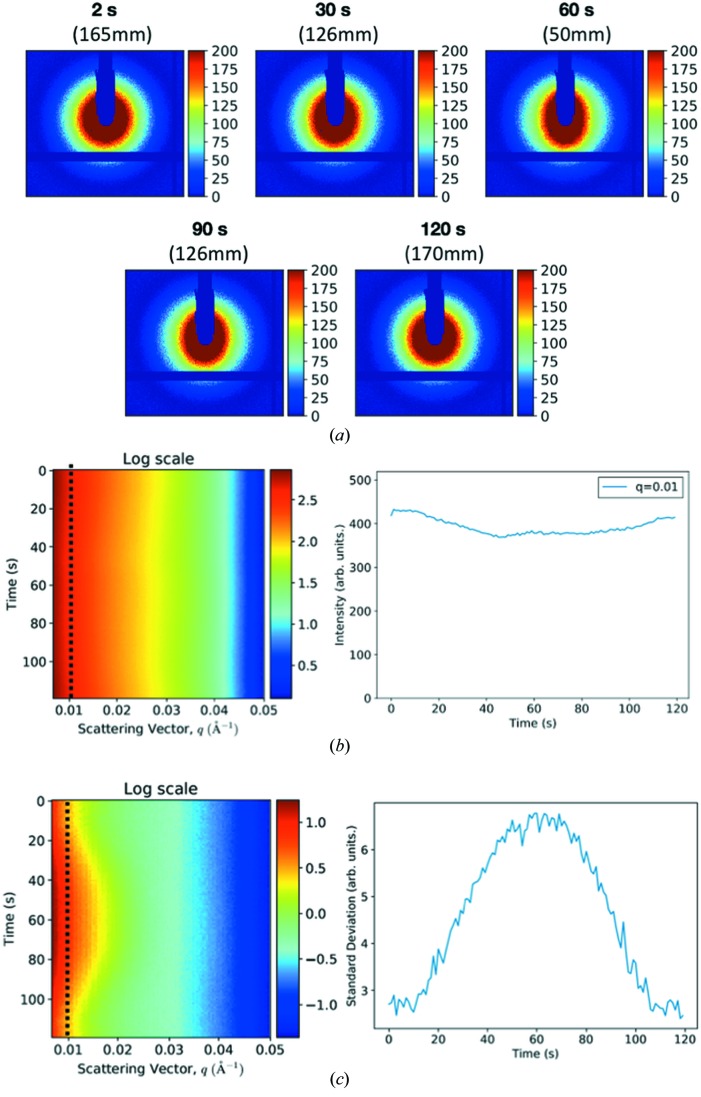
The reversible dynamics induced by the external MF. (*a*) Experimental scattering patterns at several time points, corresponding to different MF strengths that depend on the distance between the magnets. (*b*) The left panel shows the time series of the radial profile of intensity encoded in the heatmap representation and the right panel shows the time-series line profile at *q* = 0.01 Å^−1^ (indicated with a dotted line in the left panel). (*c*) The standard deviation profiles corresponding to the intensity profiles in (*b*). The left panel shows the heatmap representation and the right panel shows the standard deviation line profile at *q* = 0.01 Å^−1^.

In the time-resolved measurements designed to study the fast dynamics, the distance between magnets was changed from 170 mm to 50 mm within 50 s at a constant speed (∼2.4 mm s^−1^), paused for 20 s, and then returned to 170 mm in 50 s. Correspondingly, the MF strength was changed from 50 G to 250 G and back to 50 G in 120 s. The scattering data were measured for SMNPs at two concentrations (0.5 mg ml^−1^ and 2.0 mg ml^−1^) with varying MF. Because the results are similar at these two concentrations, we focus on the discussion of data at 2.0 mg ml^−1^. The 2D scattering patterns for SMNPs at 2.0 mg ml^−1^ concentration at several time points with the estimated position information are shown in Fig. 4 ▸(*a*) (see the supporting information for a video at more time points); the external MF strength at each time point can be obtained from the curve in Fig. 2 ▸(*c*). The radial profiles of intensity and the associated standard deviations are summarized in Figs. 4 ▸(*b*) and 4 ▸(*c*). Several interesting phenomena were observed: (1) the 2D scattering patterns became angularly anisotropic as the MF was getting stronger revealing that the elongated components (SMNP assembly/aggregates) were being aligned with the MF. Interestingly, based on the 2D patterns, the MF induced change did not occur until sufficient MF strength was reached (∼MF strength = 70.2 G at a distance of 146 mm). This is also consistent with the time series of intensity profiles and the standard deviation profiles, which are encoded in the 2D heatmaps in Figs. 4 ▸(*b*) and 4 ▸(*c*). The left panel of Fig. 4 ▸(*b*) shows the heatmap of intensity-profile time series by tiling the 120 radial intensity profiles along the *y* axis. The right panel shows the line profile at *q* = 0.01 Å^−1^. Similarly, the standard deviations associated to each radial intensity profile are represented using the heatmap in Fig. 4 ▸(*c*), and the standard deviation profile at *q* = 0.01 Å^−1^ is shown to illustrate the anisotropy levels. Fig. 4 ▸(*b*) shows that the total intensity remained at similar levels (the fluctuation range is within 10% of the average value at *q* = 0.01 Å^−1^), while Fig. 4 ▸(*c*) indicates that the anisotropy level changed significantly (the fluctuation range is nearly 80% of the average value at *q* = 0.01 Å^−1^). (2) During the period that the magnets were kept still at the closest positions, the 2D patterns showed small variations indicating that the equilibrium states were quickly achieved to adapt to the strongest MF. This corresponds to the flat region of the standard deviation time series as well as the associated line profile near *t* = 60 s (MF strength = 250 G). (3) The MF induced changes in SMNP arrangements are reversible; as the magnets were being moved apart, the scattering patterns gradually recovered to those observed at the beginning of the experiment cycle. The 2D pattern at the end of the cycle where *t* = 120 s (and the magnets returned to the starting positions) became angularly isotropic, similar to the pattern measured at the beginning of the cycle. We also noticed that the intensity was slightly reduced in the stronger MF, and recovered to the starting level when the magnets were moved apart [see Fig. 4 ▸(*b*)]. This could be attributed to the experimental setup, as a result of the detector regions that measure stronger signals being shadowed by the beamstop (see the *Discussion* section[Sec sec4] for details).

### 3.3. Components of the sample in the presence of an external MF   

**Figure 5:**
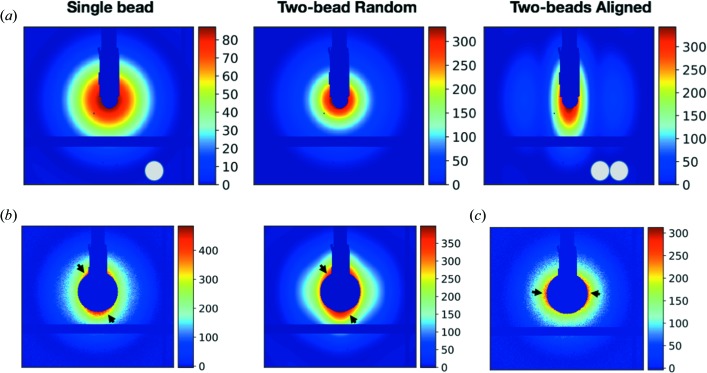
Model-based analysis of 2D scattering patterns. (*a*) Simulated 2D patterns using experimental parameters from a single SMNP and the TBC model. From left to right: scattering pattern of a single SMNP, the averaged scattering pattern from TBC particles in random orientations and the scattering pattern of the TBC particles in the aligned orientation. (*b*) The experimental data measured with the external MF. A fitted pattern created by combining simulated patterns in (*a*) with optimal weights. (*c*) Experimental data in a case where the external MF is rotated by 90°. The arrows in (*b*) and (*c*) indicate the regions with stronger scattering intensities.

Because of the external MF induced orientation preferences of the SMNP assemblies, the conventional SAXS analysis approach could not be directly applied to interpret such angularly anisotropic data. In order to quantify the components that are aligned with the external MF, simulated patterns from SMNPs and assemblies were utilized to obtain the optimal weights that best fit the 2D scattering patterns from experiments. Here, we assume that the dominant component in the assembly form is composed of two SMNPs to form a chain model, noted as the TBC model as described in the *Method* section[Sec sec2] [see Fig. 5 ▸(*a*)]. In the presence of the strongest MF the two-bead chains are aligned in the direction of the MF resulting in the changes in the 2D intensity patterns. By optimizing the composition of the particles (a single SMNP, TBC in random orientations and TBC aligned with MF), the experimental scattering pattern was matched with simulated scattering data [Fig. 5 ▸(*b*)]. The aligned TBC is about 21.6% by mass, estimated based on the percentage of three types of particles (see Table 1 ▸). Without an external MF, the percentage of aligned TBC is about 2.0%. Therefore, the MF has a strong influence on particle orientations.

In order to cross-check that the TBC particles were indeed aligned with the external MF, a control experiment was carried out by placing one magnet above the capillary, so that the new MF is perpendicular to the original one (also perpendicular to the direction of incidence X-rays). As expected, the intensity contour map shows a contracted axis that matches the TBC major axis direction [see Fig. 5 ▸(*c*)]. Using the same model-based fitting procedure (the simulated pattern for aligned TBC was rotated by 90°), the aligned TBC component in the solution was estimated to be about 9.9% by mass. This ratio is reasonable compared with the 21.6% in the MF of paired magnets because the external MF was reduced to half using only one magnet in this configuration because of the space constraint from the instruments.

## 4. Discussion and conclusion   

This is the first time the SAXS technique has been used to study the reversible dynamical rearrangement of magnetic nanoparticles in solution, to the best of our knowledge. The chain-like assemblies were observed to be aligned with the direction of the external MF. As revealed from the 2D scattering patterns and 1D radial profiles, SMNPs in solutions quickly recover to their original states after the removal of the external MF. The aligned components in solution closely depend on the MF strength: the MF needs to be strong enough to overcome thermal fluctuations so that the SMNP alignment becomes detectable. The dynamics of SMNPs also depend on the direction of the external MF, as revealed by the 2D scattering data. Applications of SMNPs and the inducing MF can be developed by varying these control parameters, based on the desired properties of assemblies.

**Figure 6:**
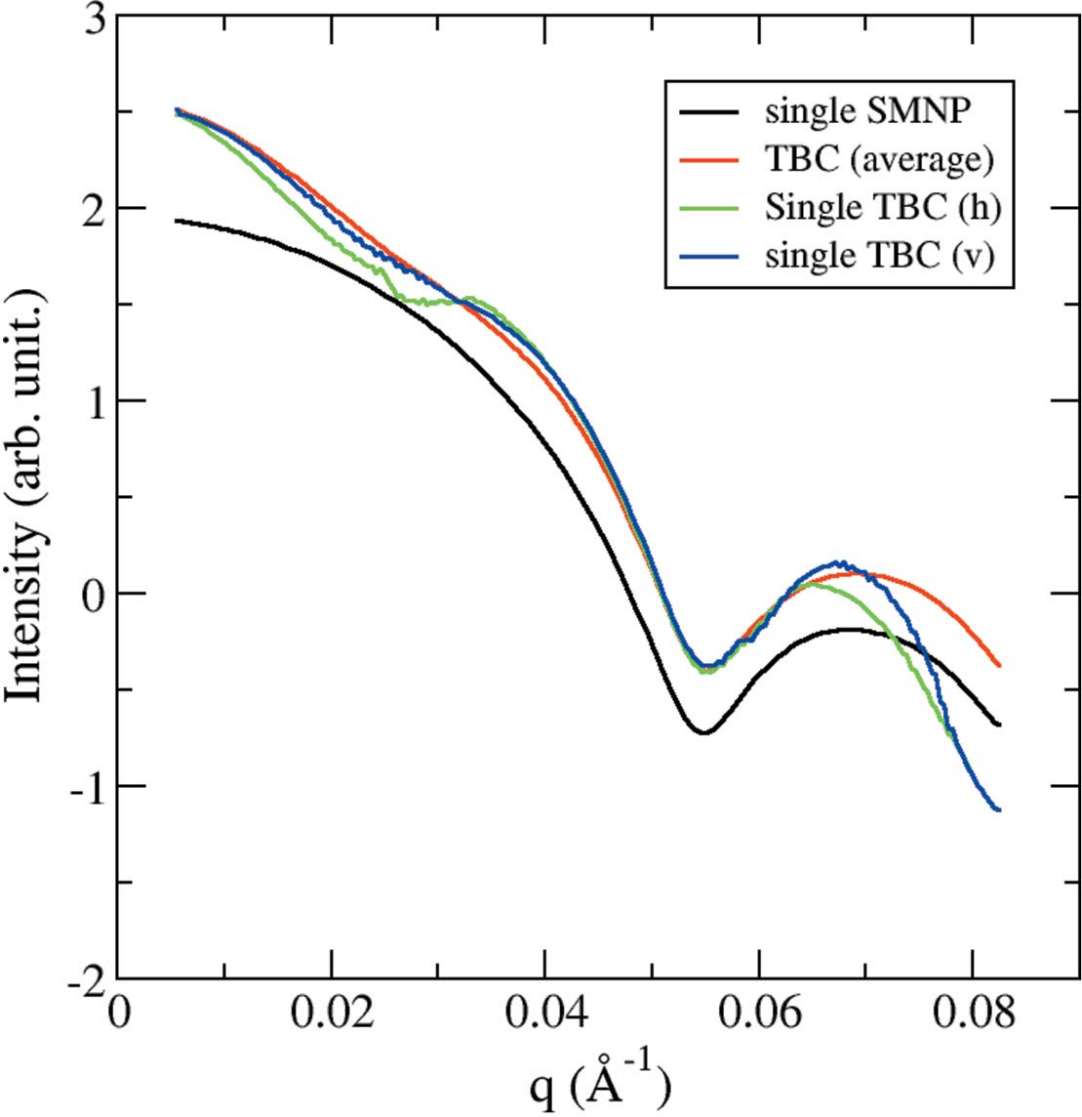
Theoretical radial profiles for the SMNP and TBC models. Each profile was calculated from simulated patterns with the same detector parameters including the beamstop shadows. The profile for a single SMNP was also included for comparison. The drop in intensity for horizontally placed TBC models is observable in the *q*-range 0.01–0.03 Å^−1^. The intensity is shown in logscale.

During the reversible process, we noticed a small drop in intensity when the external MF became stronger, as described in Section 3.2. One possibility for this is that the SMNP samples were dragged out of the X-ray focus due to the MF gradient so that the number of scattering particles became smaller. This is unlikely because the MF is nearly uniform in the exposure region for the present experimental setup as described in the *Method* section[Sec sec2]. Furthermore, because the particles are dispersive in the buffer, and the capillary was placed in the parallel direction of the applied MF, the net movement (if there is one) should be along the capillary, so that SMNPs will move across the exposure region, similar to the measurement of a flowing sample. Our understanding about this intensity drop is from the external MF induced orientation preference for the chain-like assembly, resulting in signal anisotropy. As shown in Figs. 4 ▸ and 5 ▸, the beamstop resulted in a large shadowed area on the detector, and coincidentally a stronger portion of the signals would be recorded in that region [see Fig. 4 ▸(*a*) for details]. Because the 1D SAXS data were angular averaged values, the anisotropic distribution of intensities (because of the alignment to external MF) and the unrecorded strong intensities resulted in the drop in intensity when the magnets were closer. We simulated the scattering patterns for TBC models at two orientations using the same detector parameters with the beamstop shadow, and the radial profiles are summarized in Fig. 6 ▸. It is obvious that the horizontally placed TBC particles have significantly lower intensity between *q* = 0.01 Å^−1^ and *q* = 0.03 Å^−1^ compared with the averaged intensity of randomly oriented TBC particles. The increase in intensity when magnets were moved apart also indicates that the signal anisotropy should be the cause of such variation.

In this study, the SMNPs and TBC particles were used to fit the experimental data. In reality, complicated aggregates can coexist in solution because of the fluctuations and the interactions between particles. The high-order aggregates may be studied using the pearl necklace models (Salgueiriño-Maceira *et al.*, 2006 ▸; Bonini *et al.*, 2007 ▸). At the sample concentrations used in this study (0.5–2 mg ml^−1^), each SMNP occupies a volume of approximately 10^7^ nm^3^ on average. In other words, the SMNPs are separated from each other with a distance of ∼100 nm (if they are evenly distributed). With this consideration we assume that the SMNP samples are populated in the monomer and dimer states. Single-particle scattering techniques using X-ray free-electron lasers can be applied to study the states of SMNPs in solution (Aquila *et al.*, 2015 ▸; Liu & Spence, 2016 ▸).

This study demonstrates that the time-resolved SAXS experiments can be used to provide rich information for SMNP dynamics that is not attainable using other methods. The data analysis needs special treatment of the experimental data, such as 2D image fitting, in contrast to the conventional SAXS analysis procedure, because of the orientation preference induced by the external MF. The MF induced alignment is fast, and the time-scale measurement needs finer temporal resolutions than is accessible in this experiment. In this study, the external MF was provided using permanent magnets, with the consideration to reduce heat generation. The strength was controlled by adjusting the distance between the magnets, driven by electronic motors along the supporting rail. In the interest of faster dynamics study, electromagnets can be used to generate faster variations in the external MF.

Solution-scattering experiments at synchrotron facilities have been used to study dynamics by using the pump–probe approach. The pumping can be achieved using an optical laser, a heating device or a stop-flow mixing instrument. This is the first time using an MF as the pumping medium, demonstrating that the dynamics of SMNPs can be investigated using a solution SAXS method, paving ways for studying interactions and dynamics of nanoscale particles under the influence of MFs. The method may be extended to investigate biological molecules that are susceptible to MFs.

## Supplementary Material

Supplementary video of the 2D scattering patterns for SMNP at 2 mg/ml concentration. DOI: 10.1107/S1600577519004909/co5123sup1.mp4

## Figures and Tables

**Table 1:** Weighting-factor summary for 2D scattering pattern fitting The numbers in the parentheses are the percentages measured in mass. The weighting factors are the molar percentages for a single SMNP, for TBC (randomly oriented) and for TBC (aligned to the MF). The mass percentage is derived as follows: *W*_s_/(total_mass) for a single SMNP, and 2*W*_dr_/(total_mass), 2*W*_da_/(total_mass) for TBC particles, where total_mass = *W*_s_ + 2(*W*_dr_ + *W*_da_).

Applied magnets	*W*_s_	*W*_dr_	*W*_da_
None	0.5405 (0.370)	0.4448 (0.610)	0.0147 (0.020)
Paired ring magnets (horizontal MF)	0.7197 (0.562)	0.1419 (0.222)	0.1384 (0.216)
Single ring magnet (vertical MF)	0.6548 (0.487)	0.2785 (0.414)	0.0667 (0.099)
